# News and Events of the Week

**Published:** 1911-04-01

**Authors:** 


### News and Events of the Week.

The Mayor of Hackney (Mr. Fenton Jones) unveiled a brass tablet in the Hackney Town Hall last week to the memory of the late Mr. Cox, a pioneer worker with $x$-rays. The tablet, which has been provided by residents of the borough, is inscribed: "In honour of Harry William Charles Cox, consulting electrician, who died at Hackney July 9, 1910. He contracted a malignant disease while perfecting apparatus for adapting the $x$-rays to the relief of human suffering."

The Vice-Chancellor of Manchester University (Sir Alfred Hopkinson) has announced that the University has received an anonymous gift of £1,000 for promoting research work in medical subjects.

The annual meeting of the British Medical Association will be held in Birmingham from July 25 to July 28. The president this year is Mr. H. T. Butlin, consulting surgeon to St. Bartholomew's Hospital, and the president-elect Professor Robert Saundby, professor of medicine in the University of Birmingham. The programme of business is published in a supplement to the *British Medical Journal*. The president will deliver his address on Tuesday, July 25, the address in medicine will be given on July 26 by Dr. Byron Bramwell, President of the Royal College of Physicians of Edinburgh, and the address in surgery on July 27 by Professor Jordan Lloyd, of Queen's Hospital, Birmingham. For the purposes of the scientific business of the meeting sixteen sections have been authorised by the council.

The Gillson Scholarship in Pathology, 1911, has been awarded by the Society of Apothecaries to Henry Letheby Tidy, M.A., M.B., B.Ch. Oxon, M.R.C.P. Lond., M.R.C.S., London Hospital.

The annual dinner of the staff and students of the London School of Clinical Medicine (post-graduate), Seamen's Hospital, Greenwich, was held on Friday last week at Princes' Restaurant, Piccadilly. Lord Charles Beresford presided, and the company included Sir Henry Morris (President of the Royal Society of Medicine), Reginald Talbot, Sir Dyce Duckworth, Mr. Percival Nairne (chairman of the committee), Sir William Bennett, Sir Robert Burnet, Sir Henry Dalziel, M.P., Sir Malcolm Morris, Sir T. Porter, Sir Charles McD. Cuffe, Professor R. T. Hewlett, Sir William Allchin, Mr. Charles Stonham, Dr. Squire Sprigge, and Mr. P. J. Michelli, C.M.G. (secretary). The Chairman, after announcing that the King had doubled his subscription to the Dreadnought Hospital, said that during the five years that the school had been in existence 479 students had taken out courses, and among those there were forty-seven naval surgeons. They in the Service thoroughly recognised and appreciated the work of the medical officers, and it was gratifying to know that there was such an institution to provide instruction for medical men, who, having already qualified, desired to keep themselves informed of the latest methods and discoveries in medicine and surgery. He hoped that some institution would soon be established for men in the Service who contracted consumption in the course of their duty. He referred to the question of hospital ships, and appealed for ships of that class fitted with every possible requirement. It was the duty of the State to organise their Services so that a man should not hurt his little finger while serving, much less lose his life.

In order that the standard of purity of the quinine manufactured in India may be maintained, it has been decided that all quinine issued from the Government factories shall contain not less than 95 per cent. of quinine.

The eighth International Congress for the Prevention of Tuberculosis will be held at Rome from the 24th to the 30th September, 1911. The Congress will be divided into three sections (1) the aetiology and epidemiology of tuberculosis, (2) its pathology and therapeutics, medical and surgical, (3) its prevention from a social point of view. For all information application should be made to the general secretary's offices, 36 via in Lucina, Rome.

The Paris Academy of Science has received particulars of the legacy left it by M. Loutreuil, who, though of French nationality, made a large fortune as a manufacturer in Moscow. His bequests to various learned societies and higher grade schools amount to no less a sum than 17 million francs or £680,000. Of this the Academy's share is three and a half million francs or £140,000. The interest is to be devoted to the encouragement of non-official scientists and to provide them with the funds necessary to carry out their work. It is to be administered by a special commission consisting of the president for the time being of the Academy, two permanent secretaries, and three members chosen from the sections of mathematics, natural science, and unattached academicians. A curious clause of the will may be mentioned. The donor, wishing as far as possible to assure that each legacy should remain intact, demands that the State should forgo legacy duties and be content with the already sufficiently heavy taxes of registration. In the event of this demand not being agreed to, the provisions as to the various legacies are to become null and void.

Many acts of heroism have been recorded of members of that heroic band of practitioners of all nationalities who have responded to China's appeal for help from the Powers in the matter of plague prevention. The following translation of a letter addressed to his consul at Mukden by M. Romero, French consular agent at Kharbin, describes the heroic manner in which Dr. Mesny, a French doctor, went to his death: "Kharbin, January 10, 1911. Yesterday I had the honour of informing you that I had been told of the presence here of a French doctor, but whom I had not seen. I was far from suspecting that our interview would come so soon and under such circumstances. The police having required my presence, I went to the Grand Hotel, where Dr. Mesny was staying; there a Russian doctor who had arrived told me that his colleague was attacked by the plague and that all communication with him was prohibited; but that I could talk with him in the street at a distance of two yards. I therefore waited in front of the hotel near the door out of which five minutes later our unfortunate compatriot came, surrounded by four infirmary assistants, but alert and smiling. The doctor placed me at the required distance, and M. Mesny told me that he had in all probability hardly two days to live, and that he had made his will, which he begged me to certify after his papers and effects had been disinfected; that he desired that his family should not be informed for the present, and then begged me to excuse him for not having come to see me, but that he was afraid of communicating the malady to me. He added smiling, 'I do not bid you au revoir but adieu,' and entered into the ambulance waggon which took him to the hospital. I must admit that I was touched to the point of tears at seeing a man so young, so resolute, and so courageous taking his leave so bravely.—(Signed) F. Romero."

The order made by the Battersea Borough Council on March 8 that the "brown dog" be destroyed has been carried into effect. The dog has been smashed into small pieces, and the inscription on the granite base of the memorial has been excised.

The following lectures will be delivered next week at the Central London Throat and Ear Hospital, Gray's Inn Road, W.C.: Tuesday, April 4, at 3.45 p.m., "Tracheoscopy, etc.," by Mr. J. Gay French; Friday, April 7, at 3.30 p.m., "Adenoids," by Mr. Chichele Nourse.

The following are this week's arrangements at the Medical Graduates' College and Polytechnic, 22 Chenies Street, W.C.: Monday, April 3, at 4 p.m., Dr. Wilfred Fox, Clinique (Skin); at 5.15 p.m., Dr. Nestor Tirard, "Trophic Lesions in Diabetes." Tuesday, at 4 p.m., Dr. Hector Mackenzie, Clinique (Med.); at 5.15 p.m., Dr. F. J. Poynton, "Abdominal Emergencies from the Standpoint of the Physician." Wednesday, at 4 p.m., Mr. J. M. G. Swainson, Clinique (Surg.); at 5.15 p.m., Dr. R. Maguire, "Emphysema and its Consequences." Thursday, at 4 p.m., Dr. Leonard Williams, Clinique (Med.); at 5.15 p.m., Dr. Urban Pritchard, "The Treatment of Middle Ear Suppuration." Friday, at 4 p.m., Mr. Kenneth Campbell, Clinique (Eye).

